# Reply to “Funding by Lottery: Political Problems and Research Opportunities”

**DOI:** 10.1128/mBio.01401-16

**Published:** 2016-08-30

**Authors:** Arturo Casadevall, Ferric C. Fang

**Affiliations:** a Johns Hopkins Bloomberg School of Public Health, Baltimore, Maryland, USA; b Departments of Laboratory Medicine and Microbiology, University of Washington, Seattle, Washington, USA

## REPLY

We thank Dr. Barnett for his kind comments (1). We agree that a major barrier to instituting a modified lottery is political, since government agencies are reluctant to acknowledge that peer review is unable to reliably stratify applications. It is counterintuitive that experts are little better than a random selection process at predicting the future success of proposals, even though the data are quite clear in this regard (2). Perhaps the best argument for a lottery system is that the current process is susceptible to bias. Since the publication of our commentary (3), further evidence of a systematic bias in NIH grant allocation against female applicants has been published (4). We fully agree with Dr. Barnett that more study of peer review and research funding would be useful. Funding lotteries can create new opportunities to understand the impact of funding on researchers, although we strongly suspect that a lack of funding will adversely impact productivity. We look forward to learning from the New Zealand experience.
